# Distribution Characteristics and Prognostic Value of Immune Infiltration in Oligometastatic Breast Cancer

**DOI:** 10.3389/fonc.2021.747012

**Published:** 2021-11-11

**Authors:** Danyang Zhou, Kuikui Jiang, Ruoxi Hong, Qianyi Lu, Wen Xia, Mei Li, Chengyou Zheng, Qiufan Zheng, Fei Xu, Shusen Wang

**Affiliations:** ^1^ Department of Medical Oncology, Sun Yat-Sen University Cancer Center, State Key Laboratory of Oncology in South China, Collaborative Innovation Center for Cancer Medicine, Guangzhou, China; ^2^ Department of Pathology, Sun Yat-Sen University Cancer Center, State Key Laboratory of Oncology in South China, Collaborative Innovation Center for Cancer Medicine, Guangzhou, China

**Keywords:** immune infiltration, primary tumor, oligometastatic lesion, intratumoral, peritumoral, prognostic value, oligometastatic breast cancer

## Abstract

**Background:**

To assess the distribution characteristics and the prognostic value of immune infiltration in female oligometastatic breast cancer patients.

**Methods:**

We retrospectively analyzed the clinicopathological data of oligometastatic breast cancer (OMBC) patients diagnosed between June 2000 and January 2020. Immune markers were quantified by immunohistochemistry on FFPE tissues in paired normal breast tissues, primary breast cancers and oligometastatic lesions. Survival analyses were performed using the Kaplan-Meier curves and Cox-proportional hazards model.

**Results:**

A total of 95 female OMBC patients visited Sun Yat-sen University Cancer Center between June 2000 and January 2020, and 33 of them had matched normal breast tissues, primary cancers and oligometastatic lesions and were reviewed in immune infiltration analysis. CD8 of primary tumors had a higher expression than that in matched normal tissues. The expressions of CD8 and FOXP3 were higher in the primary sites than that in the oligometastatic lesions. CD3, CD4 and CD8 were significantly lower in the intratumoral regions than that in the peritumoral regions both in primary and oligometastatic lesions. Notably, the high percentage of CD3 in the intratumoral oligometastatic lesions predicted the longer PFS and OS, and higher CD4 in the same lesions also predicted a better OS. There was obviously positive correlation between CD4/CD3 and Ki-67 in primary cancers and negative correlation between CD4/CD3 and ER in oligometastatic sites.

**Conclusion:**

We explored immune distribution and evolution in time and space in OMBC to provide new understandings for biological behaviors of this disease and further divided patients in different prognosis.

## Introduction

Breast cancer remains the most commonly diagnosed female malignant tumor with the highest incidence and mortality in 2020 worldwide (1). Distant metastasis/recurrence and its complications are the main cause of breast cancer-specific mortality. Approximately 20-30% of breast cancer patients may occur metastases after diagnosis and primary tumor treatment (2, 3), and the 5-year overall survival (OS) rate of metastatic breast cancer (MBC) patients is only 25% (4). MBC is heterogeneous both biologically and clinically in terms of proclivity for certain sites and disease burden (5). The oligometastatic breast cancer (OMBC) represents a special condition (6) and develops in about 1-10% of new MBC (7, 8). Oligometastatic disease, as a low volume metastatic disease, is defined as a state with limited number and size of metastatic lesions (up to five for breast cancer) in the 4th ESO-ESMO International Consensus Guidelines for Advanced Breast Cancer (ABC4) (9). With the improvement of the insights of oligometastasis, this disease is further classified into induced/genuine oligometastatic disease, repeat/de-novo oligometastatic disease and synchronous/metachronous oligometastatic disease (10). Due to the potential curability, oligometastatic disease aims to achieve a complete remission status and a long-term patients’ survival (11, 12). However, since no biomarker for the identification of patients with different prognoses is clinically available, the evaluation of oligometastatic disease is based solely on imaging findings and this manifestation on imaging could represent different clinical scenarios and might require different treatment strategies.

Although breast cancer is long considered as a poorly immunogenic cancer (13), the immune system plays a pivotal role in growth and development of breast cancer (14). Immunosurveillance provides an important first defense against tumor cells, on the other hand, immune responses can also lead to tumor progression by impairing tissue microenvironments and accumulating virulent cells through immunoediting (15, 16). The quantitative and qualitative differences of tumor-infiltrating lymphocytes (TILs) are associated with breast cancer progression and survival (17, 18). The high percentage of CD3+ T cells is related to better outcomes by inducing a more robust antigen-experienced, antitumor immune response (19). CD4+ T cells are divided into CD4+ T-helper 1 (Th1) cells and CD4+ T-helper 2 (Th2) cells, the former facilitates antigen presentation and predicts favorable prognoses (20), while the later inhibits cytotoxic T lymphocytes (CTLs) function, promotes an anti-inflammatory immune response, and enhances tumor growth (21). CD8+ CTLs are essential for tumor destruction. Furthermore, the immune contextures of the different compartments also have a correlation to their potential function and clinical effect. Differential densities of CD8+ and CD163+ cells in the intratumoral and peritumoral compartments are found to have significant prognostic value for clinical outcomes (22). In addition, programmed cell death protein 1 (PD-1), programmed cell death ligand 1 (PD-L1) and cytotoxic T-lymphocyte-associated protein 4 (CTLA-4), as immunotherapeutic targets, have also attracted much attention (23–25).

The relatively limited extension of disease suggests that appropriate treatment strategies can potentially cure these OMBC patients. However, the identification of reliable predictive markers able to stratify patients with different prognosis is still a challenge. The characterization of host immunity is closely related to the clinical effectiveness and prognosis of breast cancer. Monitoring immune responses in matched normal breast tissues and tumor lesions to follow their evolution along the disease progression may allow the identification of biomarkers potentially indicative of the different clinical outcomes. Therefore, to give new insights and improve the prognostic stratification, we analyzed the distribution characteristics and prognostic value of immune markers in matched normal breast tissue, primary tumor and metastatic lesions for OMBC.

## Materials and Methods

### Patient Population

Patients with breast cancer at Sun Yat-sen University Cancer Center between June 2000 and January 2020 were retrospectively reviewed. Inclusion criteria were as follows: female breast cancer patients with histologically confirmed diagnosis; metastatic disease diagnosed by pathology; no more than 5 metastatic lesions identified by imaging, including contrast-enhanced computed tomography (CT) and/or magnetic resonance imaging (MRI) and/or positron emission tomography/computed tomography (PET/CT); patients with sufficient pathological tissue to perform immunohistochemistry (IHC). Exclusion criteria were as follows: any malignancies besides breast cancer; evidences of hematological or autoimmune diseases; receipt of immune-related drugs within 3 months before tumor biopsy; induced oligometastatic disease (patients with a history of polymetastatic disease); or repeat oligometastatic disease (patients with a previous diagnosis of oligometastatic disease). Clinicopathologic information was retrieved from medical records, including age, TNM stage of primary disease, the time from primary disease to oligometastasis, oligometastatic sites and treatment strategy (including local and systemic therapy) for OMBC, and pathologic analysis of primary and oligometastatic lesions. Oligometastatic disease was defined as a situation in which disease occurred in no more than 5 metastatic sites and this state lasted for more than 6 months (the patients included was a relatively strict oligometastatic status rather than a pre stage of poly-metastasis). Progression free survival (PFS) and OS were defined as time from diagnosis of oligometastasis to the disease progression and to death (all causes), respectively. All patients were followed-up until death or study data cutoff (May 2021). The study was approved by the Ethical Committees of Sun Yat-sen University Cancer Center (NO.: B2020-319-01) and individual consent for this retrospective analysis was waived.

### Immune Assessment by Immunohistochemistry

The expression of immune markers (PD-1, PD-L1, CTLA4, CD3, CD4, CD8, FOXP3, CD68 and CD163) was quantified by immunohistochemistry on formalin-fixed paraffin-embedded (FFPE) tissues in paired patient-matched normal breast tissue, primary tumor and oligometastatic lesions. Consecutive 4-μm tissue sections were cut from blocks selected for the presence of representative tumor tissue and immunohistochemistry staining was performed in one batch per marker to prevent intensity differences. The expression of PD-1 was assessed for tumor cell and the CD68 and CD163 double-stained cells were considered as M2 tumor-associated macrophages (M2-like TAMs). CD3, CD4, CD8, FOXP3, PD-L1 and CTLA4 expression was quantified for lymphocytes in normal breast tissue and the primary/metastatic lesions, the latter were divided into the peritumoral and intratumoral by hematoxylin-eosin stain. All markers staining was reported as the percentages of positive cells per slide. The percentages were averaged from two observers and used as the final score for every sample. The two observers discussed the results to reach a consensus if there was a discrepancy (>20% difference in score).

Immunohistochemical investigations were conducted according to the standard streptavidin-biotin-peroxidase complex method. Paraffin-embedded, formalin fixed sections were dewaxed with xylene, rehydrated by graded ethanol, rinsed using deionized water, and then blocked with 3% hydrogen peroxide for 10 min at room temperature. Antigen retrieval was performed by high-pressure-cooking the samples in a 10 mM citrate buffer (pH 6.0) for 4 min. Slides were blocked with 5% normal goat serum for 30 min at room temperature and subsequently incubated with primary antibody at 4°C overnight. The primary antibodies (anti-PD-1, clone UMAB199, OriGene Technologies; anti-PD-L1, clone E1L3N, Cell Signaling Technology; anti-CTLA4, clone UMAB249, OriGene Technologies; anti-CD3, clone LN10, OriGene Technologies; anti-CD4, clone EP204, OriGene Technologies; anti-CD8, clone SP16, OriGene Technologies; anti-FOXP3, clone UMAB248, OriGene Technologies; anti-CD68, clone KP1, OriGene Technologies; anti-CD163, clone 10D6, OriGene Technologies) were diluted following manufacturer’s protocols. Secondary goat anti-mouse/rabbit antibodies (PV-6000, OriGene Technologies) were used to detect primary antibodies. The sections were counterstained with hematoxylin.

### Statistical Analysis

The continuous variables were described by median and range and the categorical variables were showed with percentages. The cutoff values for immune markers were recommended by Xtile. The median value was as the cut-off value if no appropriate cutoff value was proposed by Xtile. Spearman’s correlation coefficient or Chi-square test’s Phi coefficient served to assess the correlation among the investigated markers. The Wilcoxon signed-rank test and Kruskal-Wallis One-Way ANOVA were used for the statistical analysis of variation in immune infiltration data between different tissues. The impact of the extent of immune infiltration on PFS and OS was calculated by Kaplan-Meier curves. The Cox-proportional hazards model was carried out to evaluate the simultaneous influence on PFS and OS of all covariates. For all tests, *P* values less than 0.05 were considered statistical difference, and all *P* values were tested two sided. Statistical analysis was performed using Statistical Package for the Social Sciences (SPSS), version 25.0; Xtile, version 3.6.1 and GraphPad Prism, version 6.0.2.

## Results

### Clinicopathological Characteristics

**Figure 1:**
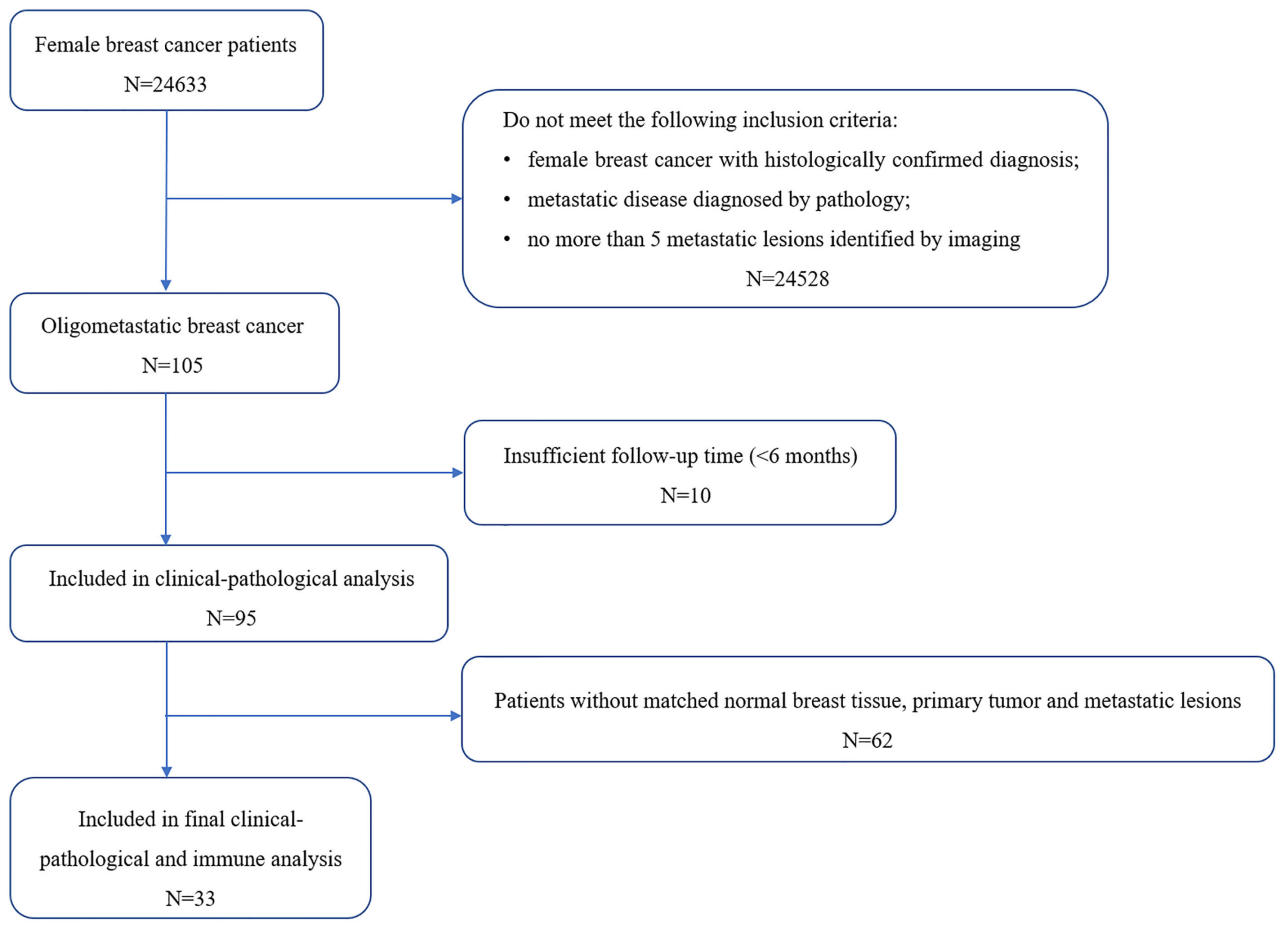
A flow chart outlining included patients’ selection.

A total of 95 female OMBC patients visited Sun Yat-sen University Cancer Center between June 2000 and January 2020, and 33 of them had matched normal breast tissues, primary cancers and oligometastatic lesions and were collected in subsequent immune infiltration analysis (**Figure 1**). The clinicopathological data of 95 patients were summarized in **Table 1**. All patients were genuine oligometastatic disease and de-novo oligometastatic disease. The pathological subtype of primary sites was all invasive ductal carcinoma (IDC). 12.6% (12/95) of the patients were synchronous oligometastatic disease, while the remaining 87.4% (83/95) were metachronous disease. Liver, lung and brain were the main oligometastatic sites, accounting for 40.0%, 29.5% and 27.4%, respectively. There were 40 hormone receptor (HR)+ (human epidermal growth factor receptor 2) HER2- breast cancer, 40 HER2+ cancers and 10 triple negative breast cancer (TNBC) patients based on the primary tumor. The median time to oligometastasis from initial diagnosis of breast cancer was 21.19 months. The median PFS and OS after oligometastatic disease were 16.73 and 162.74 months, respectively. The median follow-up time after the diagnosis of primary breast cancer was 61.0 months, and the median follow-up time after diagnosis of oligometastasis was 33.5 months. Among 95 patients included, 91 patients were performed the systemic therapy. A total of 73 (76.8%) patients received the local treatments, including surgery, radiotherapy and interventional therapy, and surgery was the main treatment strategy, accounting for 75.3%.

**Table 1:** Clinicopathological characteristics of 95 female oligometastatic breast cancer patients.

Factor	Median (range)/number (frequency)
Age at diagnosis of oligometastasis (year)	48 (25-72)
T stage (1-2/3-4/unknown)	74 (77.9%)/18 (18.9%)/3 (3.2%)
N stage (positive/negative/unknown)	73 (76.8%)/21 (22.1%)/1 (1.1%)
Molecular subtype of primary site	
(HR+HER2-/HER2+/TNBC/unknown)	40 (42.1%)/40 (42.1%)/10 (10.5%)/5 (5.3%)
Oligometastatic type (synchronous/metachronous) ^a^	12 (12.6%)/83 (87.4%)
Oligometastatic site (liver/lung/brain/others)	38 (40.0%)/28 (29.5%)/26 (27.4%)/3 (3.2%)
Molecular subtype of metastatic site	
(HR+HER2-/HER2+/TNBC/unknown)	36 (37.9%)/40 (42.1%)/12 (12.6%)/7 (7.4%)
Systemic therapy after oligometastasis ^b^ (yes/no)	91 (95.8%)/4 (4.2%)
Local treatments of oligometastatic lesions ^c^ (yes/no)	73 (76.8%)/22 (23.2%)
PFS (months)	16.73 (6.0-120.4)
OS (months)	162.74 (7.5-233.8)

a Synchronous oligometastatic disease was referred to maximum 6 months interval between diagnosis of oligometastatic disease and primary cancer diagnosis, metachronous oligometastatic disease was referred to more than 6 months interval between diagnosis of oligometastatic disease and primary cancer diagnosis.

b Among 95 patients included, 91 patients were performed the systemic therapy. 39 patients were HR+/HER2- breast cancer of primary tumor, and 20 patients were conducted the chemotherapy and 19 were carried out the endocrine therapy. 33 patients (84.6%) received anti-HER2 targeted therapy in HER2+ primary breast cancer and all 8 TNBC patients received chemotherapy.

c Local treatments of oligometastatic lesions included surgery, radiotherapy and interventional therapy.

HR, hormone receptor; HER2, human epidermal growth factor receptor 2; TNBC, triple negative breast cancer; PFS, progression free survival after oligometastasis; OS, overall survival after oligometastasis.

**Figure 2:**
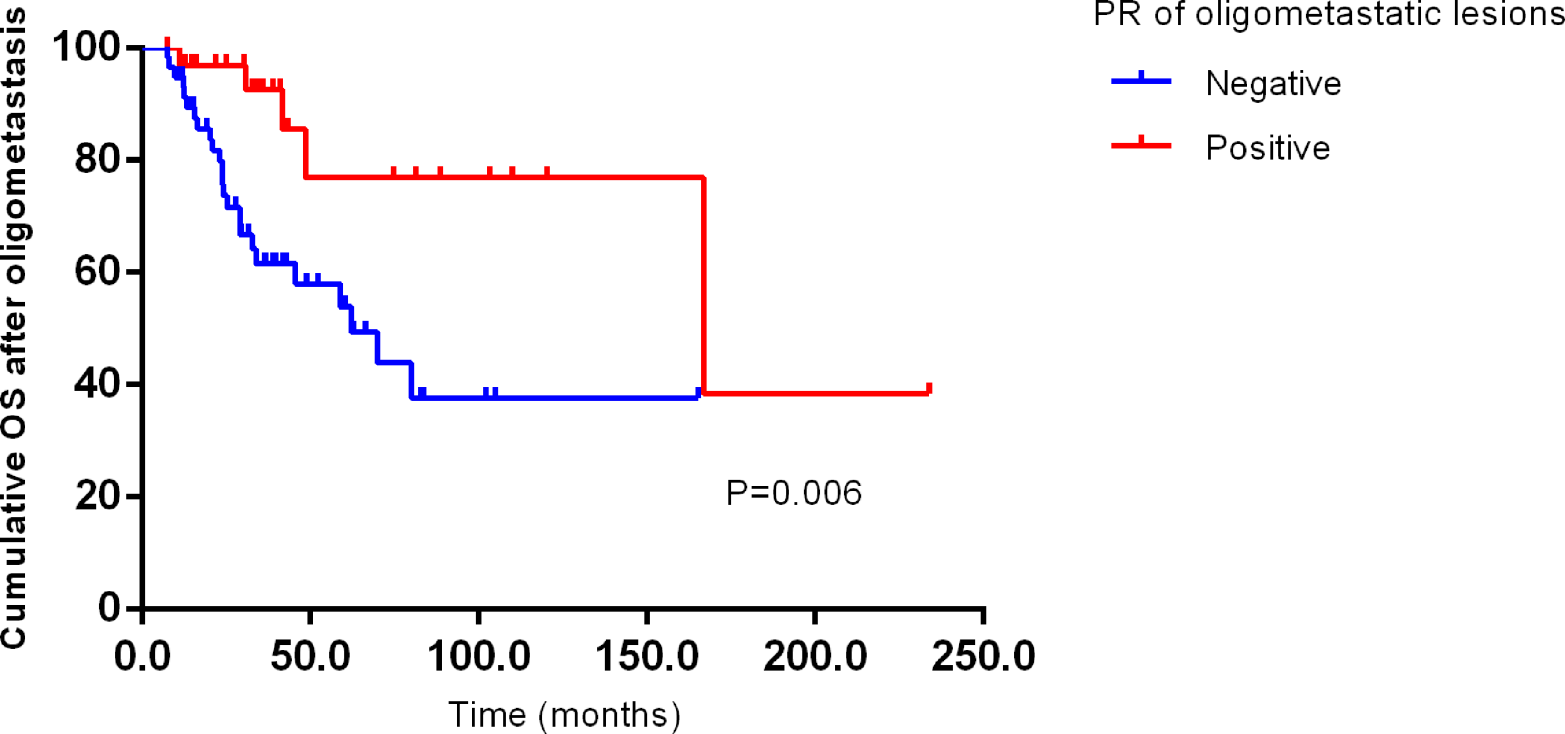
Kaplan–Meier curve for OS of oligometastatic breast cancer patients stratified by PR of oligometastatic lesions. PFS, progression free survival after oligometastasis; OS, overall survival after oligometastasis.

We further explored the impact of conventional clinicopathological factors on the survival of OMBC. Kaplan-Meier survival curves analysis suggested that progesterone receptor (PR) of oligometastatic lesions had close links with OS (*P*=0.006) (**Figure 2**), not PFS (*P*=0.734). Unfortunately, no independent impact factor was found for PFS and OS after oligometastasis on multivariate analysis (factors with *P*<0.05 and other important clinicopathological factors were included).

### Correlation Between the Clinicopathological and Immune Markers

We interrogated the correlation between the clinicopathologic factors and immune markers. Stratified of oligometastatic sites, we found that the expressions of CD3 (*P*=0.001), CD4 (*P*=0.001) and CD8 (*P*=0.011) were different in the brain, lung and liver oligometastatic samples. In the peritumoral, the expressions of CD3, CD4 and CD8 were the most abundant in liver, followed by lung and brain shown in **Supplementary Table 1**.

In primary cancer, CD4/CD3 was positively correlated with Ki-67 (the intratumoral: r=0.410, *P*=0.042; the peritumoral: r=0.414, *P*=0.029) and negatively correlated with PD-L1, but the number of positive cases of PD-L1 was small (only one sample expressed PD-L1 in the primary sites and two expressed PD-L1 in the metastatic lesions). In oligometastatic lesions, the strongest negative correlation was observed between CD4/CD3 and estrogen receptor (ER) (the intratumoral: r=-0.533, *P*=0.004; the peritumoral: r=-0.420, *P*=0.023). In addition, CD4/CD3 in peritumoral oligometastatic lesions was also inversely related to PR (r=-0.049, *P*=0.007) (**Supplementary Table 2**).

### Distribution Difference of Immune Infiltration

**Figure 3:**
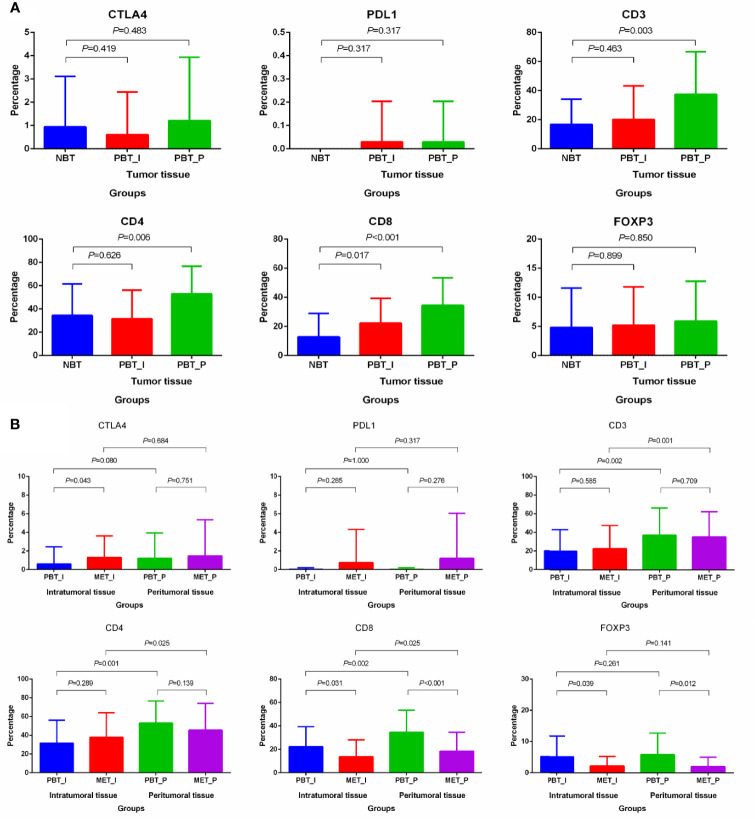
Differential distribution of immune markers in matched normal breast tissues, primary cancers and oligometastatic lesions. **(A)** Differential distribution of immune markers in normal tissue and primary lesions. **(B)** Differential distribution of immune markers in primary and metastatic lesions. NBT, normal breast tissue; PBT_I, intratumoral regions of primary breast tissue; MET_I, intratumoral regions of oligometastatic lesions; PBT_P, peritumoral regions of primary breast tissue; MET_P, peritumoral regions of oligometastatic lesions.

The different distribution of infiltrating immune cells among matched tissues and matched regions were shown in **Figure 3**. No tumor cells expressed PD-1 either in primary or metastatic lesions. The expression of CD8 (the intratumoral *P*=0.017, the peritumoral *P*<0.001) in primary sites and CD3 (*P*=0.003) and CD4 (*P*=0.004) in the peritumoral primary sites were higher than that in normal breast cancer (**Figure 3A**). For paired tumor tissues (**Figure 3B**), the percentage of intratumoral CTLA4 was higher in oligometastatic lesions than primary tumor (*P*=0.043). The higher expression of CD8 and FOXP3 were in primary breast tumors than that in oligometastatic sites both in the intratumoral and peritumoral (primary vs oligometastatic tissue: CD8: the intratumoral *P*=0.031, the peritumoral *P*<0.001; FOXP3: the intratumoral *P*=0.039, the peritumoral *P*=0.012). Further, we also compared the distribution of intratumoral and peritumoral immune infiltrating cells. For primary and oligometastatic tissue, CD3, CD4 and CD8 were less in the intratumoral than that in the peritumoral (intratumoral vs peritumoral tissue: CD3: the primary *P*=0.002, the metastatic *P*=0.001; CD4: the primary *P*=0.001, the metastatic *P*=0.025; CD8: the primary *P*=0.002, the metastatic *P*=0.025). The expression of PD-L1 and M2-like TAMs in these two regions were not significant difference both in primary and metastatic tissue. CD68 single positive cells was different (*P*=0.034) in primary and oligometastatic lesions.

**Figure 4:**
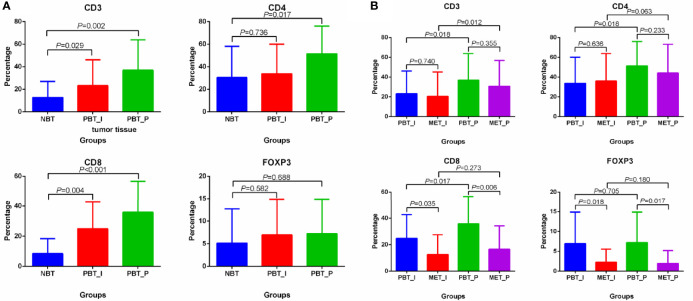
Differential distribution of CD3, CD4, CD8 and FOXP3 in matched normal breast tissues, primary cancers and oligometastatic lesions in TNBC and HER2+ breast cancer. **(A)** Differential distribution of CD3, CD4, CD8 and FOXP3 in normal tissue and primary lesions. **(B)** Differential distribution of CD3, CD4, CD8 and FOXP3 in primary and metastatic lesions. NBT, normal breast tissue; PBT_I, intratumoral regions of primary breast tissue; MET_I, intratumoral regions of oligometastatic lesions; PBT_P, peritumoral regions of primary breast tissue; MET_P, peritumoral regions of oligometastatic lesions.

Considering the close relationship between TNBC/HER2+ breast cancer and immune microenvironment, we performed the subgroup analysis of TNBC and HER2+ breast cancer. There were 19 TNBC (n=5) and HER2+ (n=14) breast cancer patients in 33 patients with immune analysis. In TNBC and HER2+ subgroup, the distribution differences of immune indexes were mainly concentrated in CD3, CD4, CD8 and FOXP3, and the characteristics was similar to the total population (**Figure 4**). The higher percentages of CD3 and CD8 in primary sites and CD4 in the peritumoral primary sites were found than that in normal breast cancer. CD8 and FOXP3 were higher in primary breast tumors than that in oligometastatic sites. In primary and oligometastatic tissue, CD3 were less from the intratumoral than that from the peritumoral.

### Prognostic Value of Immune Markers

The prognostic values of immune markers for OS and PFS in all 33 oligometastatic breast cancer patients were shown in **Figure 4**. The PFS rates were 47% at 1 year, 28% at 2 years, and 23% at 3 years; corresponding OS rates were 88%, 84%, and 78%. The median PFS for all 33 patients was 17.24 months, and the median OS was 162.00 months, which was similar to that of the overall 99 OMBC patients.

**Figure 5:**
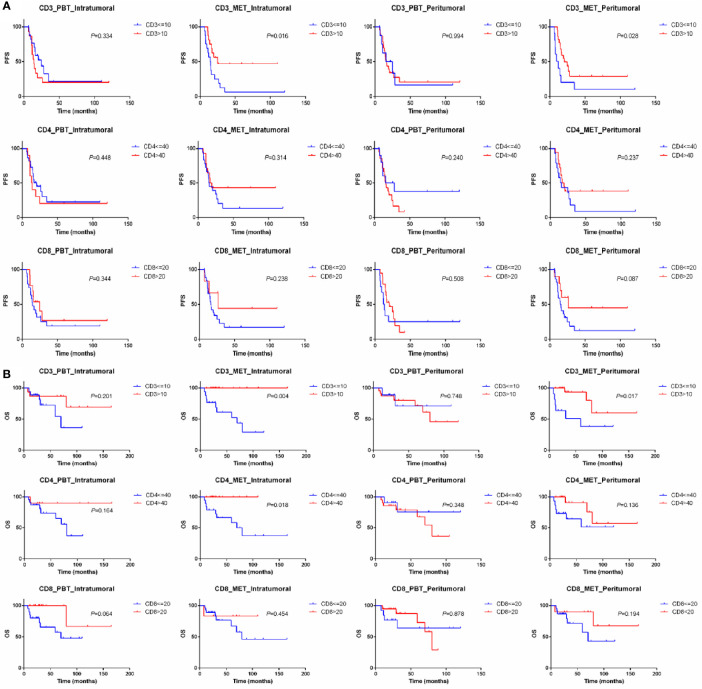
Kaplan–Meier curve for PFS **(A)** and OS **(B)** of oligometastatic breast cancer patients stratified by the expression of CD3, CD4 and CD8. PBT, primary breast tissue; MET, oligometastatic lesions.

Patients with low percentage of CD3+ immune cells in the intratumoral oligometastatic lesions had worse PFS (*P*=0.016) and OS (*P*=0.004) than did those with high percentages. Similarly, there was a statistical difference that low CD3+ T cells in the peritumoral metastatic lesions also predicted worse PFS (*P*=0.028) and OS (*P*=0.017). For OS, in addition to CD3, high CD4+ immune cells in the intratumoral metastatic lesions predicted better clinical outcomes (*P*=0.018). The expression of CD3, CD4 and CD8 in normal breast tissue and primary lesions had no prognostic value for PFS and OS after oligometastasis (**Figure 5**). CTLA4, PD-L1, FOXP3+ immune cell and M2-like TAMs in 3 types of matched tissues did not predict the clinical outcomes in OMBC patients. CD68 or CD163 single positive cells had no prognostic value in these patients.

**Figure 6:**
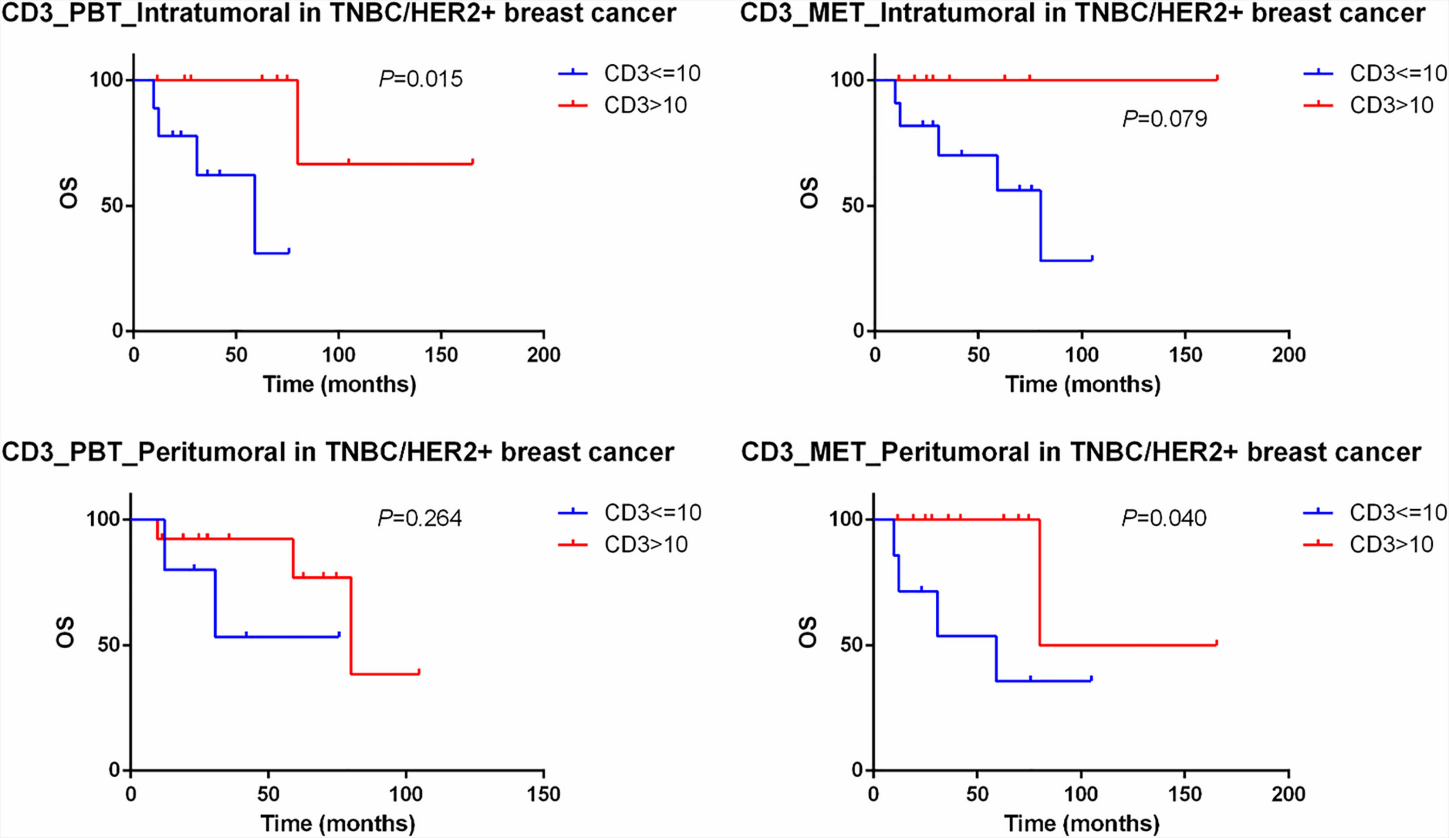
Kaplan–Meier curve for OS of TNBC and HER2+ oligometastatic breast cancer patients stratified by the expression of CD3. PBT, primary breast tissue; MET, oligometastatic lesions.

In the subgroup analysis of 19 TNBC and HER2+ breast cancer, CD3 still maintained its predictive value and the low expression of CD3 in the intratumoral primary lesions (*P*=0.015) and peritumoral oligometastatic lesions (*P*=0.040) had worse OS than did those with high expression (**Figure 6**).

## Discussion

It is now well appreciated that immune microenvironment plays a critical role in the evolution of breast cancer. Oligometastatic tumors, as a potentially curable state, are given more attention recently. Studies of the breast tumor microenvironment have largely focused on tumor mutational and transcriptional landscapes in primary and conventional polymetastatic breast cancers (26). Our study is novel in two main regards: (1) we examined three cohorts of matched normal tissues, primary breast cancers and oligometastatic lesions, allowing us to discern immune changes in the whole evolution of OMBC and (2) we further divided the same samples into the intratumoral and peritumoral regions to refine the distribution of immune infiltration in the different areas. We explored the changes of immune infiltration in time and space to extend the current cognition of OMBC and to increase the prognosis stratification, and hoped to provide reference for individual therapy.

We detected large-scale differences in the immune microenvironment in the paired primary and oligometastatic lesions, as well as normal breast tissue and primary breast cancer. Most of the markers expressed in immune cells were lower in oligometastases compared with primary tumors in varying degrees, which was consistent with previous studies on polymetastases (27, 28). Of them, the expressions of CD8 and FOXP3 were substantially lower in the oligometastatic sites than that in the primary sites both in the intratumoral and peritumoral regions. Cytotoxic T cells, identifiable by CD8 expression, recognize cells that present foreign antigens in association with the major histocompatibility complex class I molecule through a specific interaction between the presented antigen and the T-cell receptor (29). Cytotoxic T cells, as a major effector component of the adaptive immune system, can act on tumor cells which can present atypical antigens (30, 31), which also partly explained why the CD8 of the primary lesions was higher than that of the normal tissues. Regulatory T cells, which express FOXP3, promote tumor growth by inducing host tolerance against tumor antigens by attenuating the T cell-mediated immune response against the tumor cells and enabling them to evade the antitumor immune response (32). Although these two functionally distinct subsets of T cells exerted paradoxical effects in immune response, the expression CD8 and FOXP3 depleted probably due to a decrease in the overall immune level in oligometastatic lesions. In terms of the regional distribution of immune infiltration, CD3, CD4 and CD8 were significantly lower in the intratumoral than that in the peritumoral both in primary and oligometastatic lesions. This implied a difference in the distribution significance of the immune cells between the intratumoral and peritumoral regions of oligometastatic breast cancer, which might be due to the difference in intensity of the immune response at the two regions (33). Colorectal liver oligometastasis was the best understood tumors on oligometastasis and the findings also showed a lower expression of immune markers (CD3, CD8 and FOXP3) and a lower TILs density in the intratumoral regions of liver metastases than in the peritumoral regions, which was in line with our results (33–35). And immunosuppression might be promoted by a high tumor burden in the intratumoral microenvironment (36, 37).

The analysis of prognostic implications of lymphocytic subsets and density demonstrated that the high percentage of CD3 in the intratumoral oligometastatic lesions predicted the longer PFS and OS, and higher expression of CD4 in the same lesions was related to a better OS. CD3 is expressed on the surface of mature T cells and is associated with better outcomes based on previous studies (38, 39). The role of CD4+ TILs in breast cancer is complex and the numbers and cell subsets of CD4+ T cells dynamically changed with breast cancer progression (40, 41). Preclinical researches showed that CD4+ T cells changed their dominant subsets from Th1 in the early stages to Treg and Th17 cells in the late stages of the cancer progression (42), interestingly, oligometastatic disease was proposed as an intermediate state between localized and systemically metastasized disease. The less specific and, perhaps, biologically irrelevant total CD3 and, occasionally, complex and dynamic CD4 density may offer prognostic information in oligometastatic setting, while the individual stromal and intratumoral lymphocytic subset markers may not so. While immunohistochemistry improves accuracy of these markers expressed on lymphoid cells to assess the clinical importance of subtyping lymphocytes, at the present time any added value from these markers is unclear (43). From the biological aspect, none of the CD3, CD4, and other immunophenotypes can be considered as a surrogate of the extreme heterogeneity and functional diversity of these lymphocytic populations in the tumor microenvironment (44). Since the immune contexture in breast carcinomas and methodological limitations, it appears that less specific markers offer more information than the more specific but still partly understood ones.

The balance among various immune cells was also worthy of attention, which reflects the immune response in the tumor microenvironment, TIL ratios may be also a predictor of clinical outcome. In our study, there was obviously positive correlation between CD4/CD3 and Ki-67 in primary cancer and negative correlation between CD4/CD3 and ER in oligometastatic cancer. The quantitative balance between different subsets of TILs is also revealed by the immune cell ratio, which may be more reliable to indicate the immunologic response status on the tumor microenvironment. Highly proliferating metastatic tumors, possibly because proliferation is related to higher levels of genomic aberrations and, therefore to produce the neoantigens, may attract T cells (45). Perhaps, adding proliferation index, as Ki-67, to the known associations between immune infiltration and subtypes may expand the knowledge of characterizing the status of host anti-tumor immune response, which needs to be taken into account in breast cancer therapeutics. Hormone receptor positive tumors have less TIL. The decreased lymphocytic infiltrate may be due to the expression of the estrogen receptor which has been shown to both promote a Th2 immune environment and decrease MHC class II expression in breast cancer cells (46, 47).

Considering different types of oligometastatic disease, all 95 OMBC patients included were genuine oligometastatic disease and de-novo oligometastatic disease, which reduced the heterogeneity of the population in terms of biological behavior and drug response of this disease (10). We did not further classify OMBC patients with simultaneous and metachronous metastases for the following reasons: 1) No consensus approached about the interval between diagnosis of primary cancer and oligometastasis to differentiate between synchronous and metachronous disease, especially synchronous disease (48). 2) The view that synchronous oligometastatic disease was associated with a worse prognosis than metachronous oligometastatic disease (49) were not confirmed by all studies (50). In addition, we explored to add time index, namely the time from diagnosis of oligometastatic disease to disease progression more than 6 months, to the current definition of oligometastasis to ensure a relatively strict oligo-metastatic status rather than a pre stage of poly-metastasis.

Our study also has several limitations. This is a small retrospective study of patient-matched pairs of primary and oligometastatic tumor samples from breast cancer. Our results should be interpreted with caution and a larger number of OMBC are needed to test the strength of our findings. Second, the receipt of other treatments before biopsy of oligometastasis, such as surgery, radiotherapy and chemotherapy, may have influenced the expression of immune cells in our patients. Third, the gene and RNA test were not carried out due to the limitation of specimen. The implementation of multiomics analysis can well explain the difference of immune infiltration in the multiple level. Despite these limitations, our study clearly highlights on the evolution and involvement of immune infiltration in the progression from a primary tumor to its oligometastatic cascade in breast cancer patient. In addition, we shed light on the prognostic values of immune markers and provided new insights for biological behaviors of the disease and further individualized treatment in OMBC.

Increasing attention has been paid to oligometastatic tumors due to the potentially curable possibility. We discerned immune changes in the whole evolution of OMBC and further refined the distribution of immune infiltration in the different regions. In addition, we found that high expression of CD3 in the intratumoral oligometastatic lesions predicted the longer PFS and OS. We improved the stratification of prognosis and provided new insights for biological behaviors of the disease and further individualized treatment in OMBC patients.

## Data Availability Statement

The raw data supporting the conclusions of this article will be made available by the authors, without undue reservation.

## Ethics Statement

The studies involving human participants were reviewed and approved by the Ethical Committees of Sun Yat-Sen University Cancer Center (NO.: B2020-319-01). The patients/participants provided their written informed consent to participate in this study.

## Author Contributions

Conception and design: DZ, KJ, FX, and SW. Administrative support: FX and SW. Provision of study materials or patients: DZ, KJ, RH, QL, and WX. Collection and assembly of data: DZ, KJ, RH, QL, and WX. Data analysis and interpretation: DZ, KJ, ML, CZ, and QZ. Manuscript writing: all authors. Final approval of manuscript: all authors.

## Funding

The study was funded by National Natural Science Foundation of China U1601224, National Natural Science Foundation of China 81272896, and National Natural Science Foundation of China 81602313.

## Conflict of Interest

The authors declare that the research was conducted in the absence of any commercial or financial relationships that could be construed as a potential conflict of interest.

The reviewer CG declared a shared affiliation with the authors to the handling editor at the time of the review.

## Publisher’s Note

All claims expressed in this article are solely those of the authors and do not necessarily represent those of their affiliated organizations, or those of the publisher, the editors and the reviewers. Any product that may be evaluated in this article, or claim that may be made by its manufacturer, is not guaranteed or endorsed by the publisher.

## Abbreviations

CTLA-4, cytotoxic T-lymphocyte-associated protein 4; CTLs, cytotoxic T lymphocytes; ER, estrogen receptor; FFPE, formalin-fixed paraffin-embedded; IDC, invasive ductal carcinoma; IHC, immunohistochemistry; MBC, metastatic breast cancer; OMBC, oligometastatic breast cancer; OS, overall survival; PD-1, programmed cell death protein 1; PD-L1, programmed cell death ligand 1; PFS, progression free survival; PR, progesterone receptor; SPSS, Statistical Package for the Social Sciences; TAMs, tumor-associated macrophages; Th1, T-helper 1; Th2, T-helper 2; TILs, tumor-infiltrating lymphocytes.
