# Supplementary material for: Distribution Characteristics and Prognostic Value of Immune Infiltration in Oligometastatic Breast Cancer
Source: Front Oncol. 2021 Nov 11;11:747012. doi: 10.3389/fonc.2021.747012 (PMC8632540; doi:10.3389/fonc.2021.747012)
Supplement: Supplementary file 1 [file DataSheet_1.docx]

Supplement Table 1. Differential distribution of immune markers in different oligometastatic sites.

| Factor | Median (range) / number (frequency) | | | | | |
| --- | --- | --- | --- | --- | --- | --- |
| Oligometastatic sites | Brain (n=15) |  | Lung (n=10) |  | Liver (n=7) |  |
| Distribution | intratumoral | peritumoral | intratumoral | peritumoral | intratumoral | peritumoral |
| ER (>=1%) | 10 (66.7%) |  | 3 (30.0%) |  | 6 (85.7%) |  |
| PR (>=1%) | 6 (40%) |  | 1 (10.0%) |  | 4 (57.1%) |  |
| HER2 (positive) | 5 (33.3%) |  | 4 (40.0%) |  | 3 (42.9%) |  |
| Ki-67(>=15%) | 12 (80.0%) |  | 5 (50.0%) |  | 5 (71.4%) |  |
| CTLA4 | 0 (0-2) | 0 (0-20) | 0 (0-10) | 0 (0-10) | 2 (0-5) | 2 (0-5) |
| PD-L1 | 0 (0-0) | 0 (0-0) | 0 (0-5) | 0 (0-20) | 0 (0-20) | 0 (0-20) |
| CD3 | 10 (0-50) | 10 (0-50) | 16.5 (0-70) | 35 (0-70) | 30 (1-70) | 70 (50-80) |
| CD4 | 30 (0-60) | 20 (0-70) | 45 (5-70) | 45 (3-80) | 70 (1-80) | 80 (60-80) |
| CD8 | 5 (0-30) | 8 (1-50) | 15 (3-50) | 10 (3-50) | 20 (1-60) | 30 (20-60) |
| FOXP3 | 1 (0-5) | 1 (0-5) | 0.5 (0-10) | 0.5 (0-10) | 0 (0-10) | 0 (0-10) |
| CD68CD163 | 50 (1-80) |  | 60 (5-100) |  | 70 (30-80) |  |
| CD4/CD3 | 1.3 (0.3-10.0) | 1.2 (0.0-4.0) | 1.7 (0.8-40.0) | 1.3 (0.6-26.7) | 1.3 (0.5-10.0) | 1.0 (1.0-1.6) |
| CD8/CD3 | 0.3 (0.0-5.0) | 0.5 (0.0-3.0) | 0.7 (0.1-30.0) | 0.6 (0.1-5.0) | 0.5 (0.2-1.0) | 0.5 (0.3-0.9) |
| CD4/CD8 | 3.0 (0.0-50.0) | 2.0 (0.0-50.0) | 1.6 (1.0-20.0) | 3.5 (1.0-26.7) | 3.5 (1.0-12.0) | 2.0 (1.2-4.0) |
| FOXP3/CD3 | 0.1 (0.0-1.0) | 0.1 (0.0-0.5) | 0.0 (0.0-5.0) | 0.0 (0.0-0.3) | 0.0 (0.0-1.5) | 0.0 (0.0-0.1) |

Note: One patient's oligometastatic site was adrenal gland.

ER, PR, HER2 and Ki-67 referred to the expression of ER, PR, HER2 and Ki-67 at the oligometastatic sites, respectively.

No expression of PD-L1 was found in oligometastatic brain site.

The CD68 and CD163 double-stained cells were considered as M2-like TAMs at the oligometastatic sites.

Abbreviations: ER, estrogen receptor; PR, progesterone receptor; HER2, human epidermal growth factor receptor 2.

Supplement Table 2. The correlation between the clinical-immune markers in different regions of primary and metastatic lesions.

1. Data from the intratumoral primary breast cancer

| ALL | ER |  | PR |  | HER2 |  | Ki-67 |  |
| --- | --- | --- | --- | --- | --- | --- | --- | --- |
|  | Corr | Corr coeff | Corr | Corr coeff | Corr | Corr coeff | Corr | Corr coeff |
| CTLA4 | 0.610 | 0.094 | 0.817 | -0.042 | 0.095 | -0.300 | 0.442 | 0.146 |
| PD-L1 | 0.295 | 0.191 | 0.264 | 0.204 | 0.417 | -0.149 | **<0.001** | -0.695 |
| CD3 | 0.090 | -0.305 | 0.197 | -0.234 | 0.149 | 0.261 | 0.435 | -0.148 |
| CD4 | 0.251 | -0.209 | 0.165 | -0.252 | 0.880 | 0.028 | 0.077 | 0.328 |
| CD8 | 0.083 | -0.311 | 0.138 | -0.268 | 0.484 | 0.128 | 0.412 | 0.155 |
| FOXP3 | 0.985 | -0.003 | 0.706 | -0.069 | 0.456 | 0.137 | 0.196 | 0.243 |
| CD4/CD3 | 0.922 | 0.020 | 0.481 | -0.142 | 0.316 | -0.200 | **0.042** | 0.410 |
| CD8/CD3 | 0.768 | -0.059 | 0.562 | -0.117 | 0.634 | -0.096 | 0.255 | 0.236 |
| CD4/CD8 | 0.627 | 0.091 | 0.907 | 0.022 | 0.769 | -0.055 | 0.330 | 0.188 |
| FOXP3/CD3 | 0.390 | 0.172 | 0.625 | 0.099 | 0.771 | -0.059 | 0.101 | 0.336 |

1. Data from the peritumoral primary breast cancer

| ALL | ER |  | PR |  | HER2 |  | Ki-67 |  |
| --- | --- | --- | --- | --- | --- | --- | --- | --- |
|  | Corr | Corr coeff | Corr | Corr coeff | Corr | Corr coeff | Corr | Corr coeff |
| CTLA4 | 0.436 | 0.143 | 0.778 | 0.049 | 0.072 | -0.322 | 0.402 | 0.159 |
| PD-L1 | 0.295 | 0.191 | 0.264 | 0.204 | 0.417 | -0.149 | **<0.001** | -0.695 |
| CD3 | 0.800 | -0.047 | 0.860 | 0.033 | 0.737 | -0.063 | 0.322 | -0.187 |
| CD4 | 0.637 | -0.088 | 0.340 | -0.177 | 0.227 | -0.223 | 0.361 | 0.173 |
| CD8 | 0.345 | -0.175 | 0.666 | -0.081 | 0.859 | -0.033 | 0.870 | -0.031 |
| FOXP3 | 0.739 | 0.062 | 0.969 | 0.007 | 0.620 | 0.093 | 0.155 | 0.266 |
| CD4/CD3 | 0.478 | -0.137 | 0.159 | -0.269 | 0.764 | -0.058 | **0.029** | 0.414 |
| CD8/CD3 | 0.562 | -0.112 | 0.421 | -0.155 | 0.932 | 0.017 | 0.512 | 0.129 |
| CD4/CD8 | 0.587 | 0.101 | 0.985 | -0.004 | 0.295 | -0.194 | 0.216 | 0.232 |
| FOXP3/CD3 | 0.875 | -0.030 | 0.510 | -0.127 | 0.391 | 0.165 | 0.139 | 0.287 |

1. Data from the intratumoral oligometastatic lesions

| ALL | ER |  | PR |  | HER2 |  | Ki-67 |  |
| --- | --- | --- | --- | --- | --- | --- | --- | --- |
|  | Corr | Corr coeff | Corr | Corr coeff | Corr | Corr coeff | Corr | Corr coeff |
| CLTA4 | 0.428 | -0.145 | 0.222 | -0.222 | 0.718 | -0.068 | 0.503 | 0.144 |
| PD-L1 | 0.751 | -0.058 | 0.611 | 0.093 | 0.260 | -0.209 | 0.771 | 0.063 |
| CD3 | 0.175 | 0.246 | 0.094 | 0.301 | 1.000 | 0.000 | 0.225 | 0.257 |
| CD4 | 0.552 | 0.109 | 0.387 | 0.158 | 0.765 | -0.056 | 0.093 | 0.350 |
| CD8 | 0.485 | 0.128 | 0.331 | 0.178 | 0.920 | -0.019 | 0.092 | 0.352 |
| FOXP3 | 0.600 | 0.096 | 0.421 | -0.147 | 0.573 | 0.105 | 0.942 | 0.016 |
| CD4/CD3 | **0.004** | -0.533 | 0.054 | -0.375 | 0.905 | -0.024 | 0.356 | 0.207 |
| CD8/CD3 | 0.286 | -0.213 | 0.555 | -0.119 | 0.904 | 0.024 | 0.114 | 0.346 |
| CD4/CD8 | 0.834 | 0.039 | 0.712 | -0.068 | 0.229 | -0.222 |  |  |
| FOXP3/CD3 | 0.912 | -0.022 | 0.699 | -0.078 | 0.285 | 0.213 | 0.107 | -0.353 |

1. Data from the peritumoral oligometastatic lesions

| ALL | ER |  | PR |  | HER2 |  | Ki-67 |  |
| --- | --- | --- | --- | --- | --- | --- | --- | --- |
|  | Corr | Corr coeff | Corr | Corr coeff | Corr | Corr coeff | Corr | Corr coeff |
| CTLA4 | 0.472 | -0.132 | 0.620 | -0.091 | 0.534 | -0.116 | 0.541 | 0.131 |
| PD-L1 | 0.717 | -0.067 | 0.644 | 0.085 | 0.260 | -0.209 | 0.770 | 0.063 |
| CD3 | 0.081 | 0.313 | 0.307 | 0.186 | 0.952 | 0.011 | 0.197 | 0.273 |
| CD4 | 0.407 | 0.152 | 0.815 | -0.043 | 0.719 | -0.067 | 0.094 | 0.350 |
| CD8 | 0.127 | 0.276 | 0.479 | 0.130 | 0.561 | -0.108 | 0.170 | 0.289 |
| FOXP3 | 0.689 | 0.074 | 0.839 | -0.037 | 0.868 | 0.031 | 1.000 | 0.000 |
| CD4/CD3 | **0.023** | -0.420 | **0.007** | -0.049 | 0.930 | -0.017 | 0.094 | 0.349 |
| CD8/CD3 | 0.589 | -0.105 | 0.982 | 0.004 | 0.296 | -0.201 | 0.672 | 0.091 |
| CD4/CD8 | 0.864 | -0.032 | 0.347 | -0.172 | 0.647 | 0.086 | 0.096 | 0.348 |
| FOXP3/CD3 | 0.832 | -0.041 | 0.751 | 0.062 | 0.717 | -0.070 | 0.211 | -0.265 |

Abbreviations: ER, estrogen receptor; PR, progesterone receptor; HER2, human epidermal growth factor receptor 2.
